# Supplementary material for: Immuno-Microbial Signature of Vaccine-Induced Immunity against SARS-CoV-2
Source: Vaccines (Basel). 2024 Jun 7;12(6):637. doi: 10.3390/vaccines12060637 (PMC11209251; doi:10.3390/vaccines12060637)
Supplement: Supplementary file 1 [file vaccines-12-00637-s001.zip › vaccines-3003388-supplementary.pdf]

Supplemental Material

Immuno-microbial signature of vaccine-induced immunity against SARS-CoV-2

Lesley Umeda [1*], Amada Torres [2*], Braden P Kunihiro [1], Noelle C Rubas [1], Riley K Wells [1], Krit Phankitnirundorn [2], Peres [2], Ruben Juarez[3,4], and Alika K. Maunakea [2, ,4**]

[1]Department of Molecular Biosciences and Bioengineering, University of Hawaii, Honolulu, HI, 96822 USA; umedal@hawaii.edu, bradenku@hawaii.edu, nrubas@hawaii.edu, rkwells@hawaii.edu,
[2]Department of Anatomy, Biochemistry, and Physiology, John A. Burns School of Medicine, University of Hawaii, Honolulu, HI, 96822 USA; torres91@hawaii.edu, peres@hawaii.edu, amaunake@hawaii.edu
[3]Department of Economics and UHERO, University of Hawaii, Honolulu, HI, USA; rubenj@hawaii.edu,
[4]Hawaii Integrated Analytics, LCC, 2800 Woodlawn Dr #141, Honolulu, HI, 96822 USA
* These authors contributed equally
**Correspondence: amaunake@hawaii.edu; Tel.: (808) 956-9282

Supplementary Figure S1. Characteristics of the participants.
Supplementary Figure S2. Temporal changes in anti-SARS-CoV-2 antibody levels.
Supplementary Figure S3. Temporal changes in relative abundance of gut microbiota.
Supplementary Table S1. Gut microbiota differences between neutralization groups.

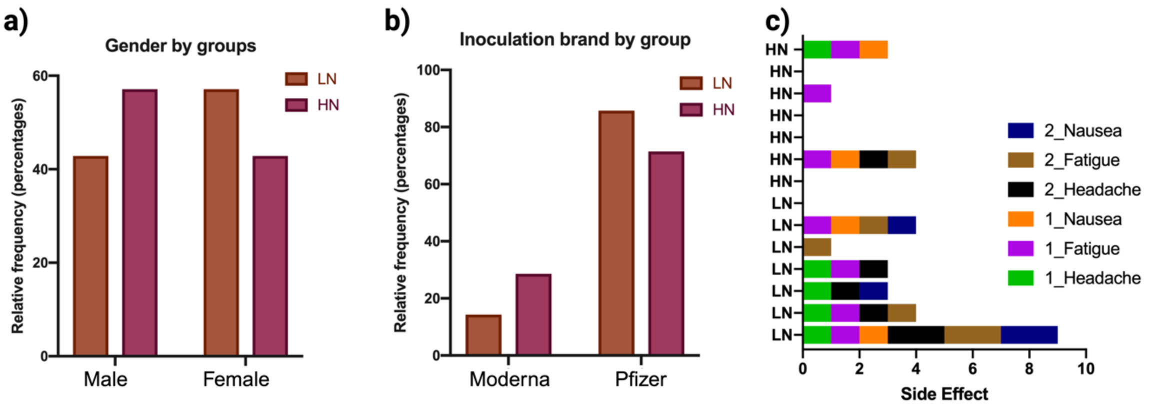

Supplementary Figure S1. Characteristics of the participants. **a**) Bar graph showing no statistical differences in the number (%) of male and female participants by high (HN) or low (LN) neutralization group. **b**) Bar graph showing no statistical differences in the number (%) of vaccine brands used by participants per neutralization group; Pfizer (BNT162b2, Pfizer-BioNTech COVID-19) and Moderna (COVID-19 Vaccine SPIKEVAX, monovalent or Bivalent). **c**) Frequency of side effects reported by participants after inoculation with each bar representing an individual participant. Side effects experienced by participants during the study include fatigue (86%), headache (57%), nausea (36%), chills (29%), fever (15%), body aches (7%), runny nose (7%), and cough (7%). The symptom is represented as "1" after the first dose, and 2 after the second dose of the vaccine.

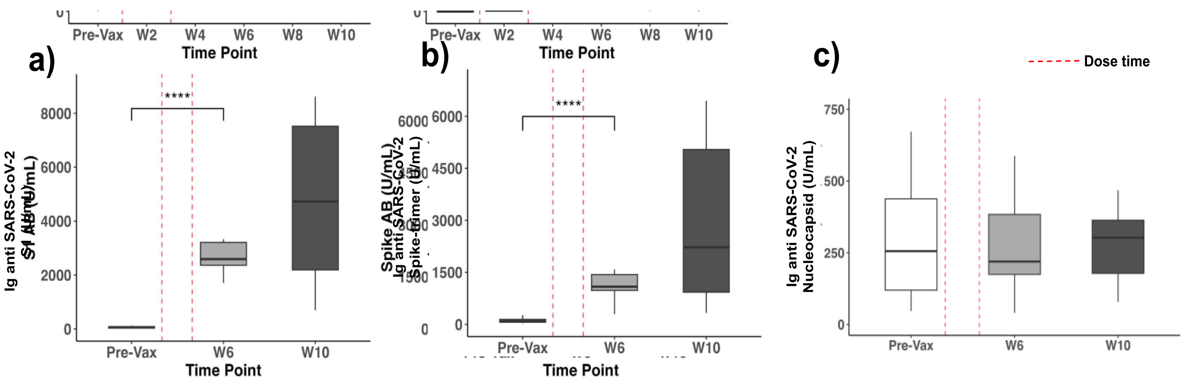

Supplementary Figure S2. Temporal changes in anti-SARS-CoV-2 antibody levels. Box plots of overall mean plasma levels of the indicated anti-SARS-CoV-2 antibody (Ig total: IgG, IgM, and IgA) for all individuals in the cohort from pre-vaccination (pre-vax), 6 weeks (W6) and 10 weeks (W10) post-vaccination of against various regions of the trimeric Spike protein with **a**) S1 and **b**) Spike-trimer showing significant differences in antibody levels pre- and post-vaccination, while **c**) the levels of Nucleocapsid antibodies showed no significant difference over the timepoints, as expected. The dotted red line represents the approximate times of vaccination. Kruskal-Wallis and *post hoc* Dunn's for multiple comparison tests were performed on group means to determine significance level at $p \leq 0.0001$.

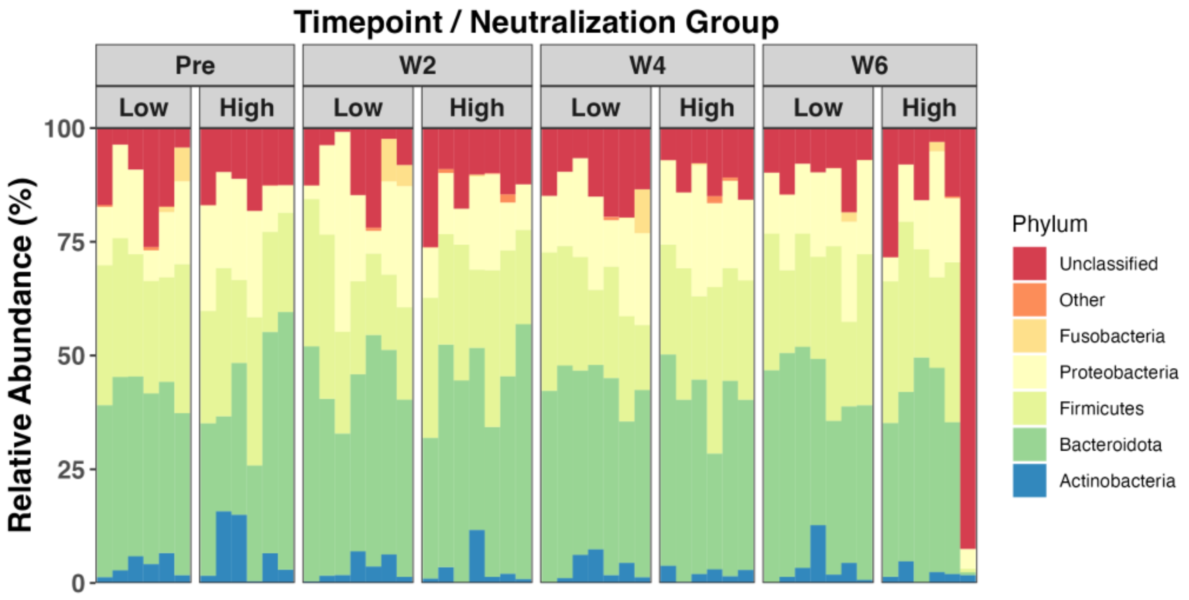

Supplementary Figure S3. Temporal changes in relative abundance of gut microbiota. Each column represents an individual participant's changes in the relative abundance at the phylum level. Low and high refer to the neutralization group over various time points before ("Pre") and after vaccination at 2, 6, and 10 weeks. Modest modifications were observed for most individuals, except for one individual in the high neutralization group at week 10. Subsampled reads on the species-level were converted to per-sample relative abundance values via the microbiome R package.

Supplementary Table S1. Gut microbiota differences between neutralization groups.

	Group Means		
**Metric**	**LN**	**HN**	***P****
**Gut Microbiota**			
**Family**			
Oxalobacteraceae	1E-4 ± 7E-5	4E-4 ± 1E-4	0.003
Prevotellaceae	2.1E-3 ± 1E-3	1.1E-2 ± 3.9E-3	0.010
**Genus**			
Clostridium	3.9E-3 ± 1E-3	8E-3 ± 1.3E-3	0.014
Coprococcus	3E-4 ± 1.3E-4	8E-4 ± 1.7E-4	0.006
Prevotella	2E-4 ± 8E-5	0 ± 0	0.003
**Species**			
Alistipes putredinis	2E-3 ± 8.9E-4	4.5E-3 ± 1.1E-3	0.013
Bacteroides fragilis	0 ± 0	2E-4 ± 9E-5	0.009
Bacteroides massiliensis	0 ± 0	2.3E-3 ± 1.1E-3	0.003
Bacteroides stercoris	9E-4 ± 4.2E-4	2.3E-3 ± 6.3E-4	0.016
Bacteroides thetaiotaomicron	5E-4 ± 1.9E-4	1.6E-3 ± 3.3E-4	0.008
Eubacterium rectale	0 ± 0	2E-4 ± 5E-5	0.021
Prevotella copri	1E-4 ± 7E-5	0 ± 0	0.012
Roseburia sp.	0 ± 0	1E-4 ± 4E-5	0.027

* Significance determined by Wilcoxon signed rank test at $p < 0.05$.
